# Gain-of-Function R225W Mutation in Human AMPKγ_3_ Causing Increased Glycogen and Decreased Triglyceride in Skeletal Muscle

**DOI:** 10.1371/journal.pone.0000903

**Published:** 2007-09-19

**Authors:** Sheila R. Costford, Nihan Kavaslar, Nadav Ahituv, Shehla N. Chaudhry, Wendy S. Schackwitz, Robert Dent, Len A. Pennacchio, Ruth McPherson, Mary-Ellen Harper

**Affiliations:** 1 Department of Biochemistry, Microbiology and Immunology, Faculty of Medicine, University of Ottawa, Ottawa, Ontario, Canada; 2 Division of Cardiology and Lipoprotein and Atherosclerosis Research Group, University of Ottawa Heart Institute, Ottawa, Ontario, Canada; 3 Ottawa Hospital Weight Management Clinic, Ottawa, Ontario, Canada; 4 Genomics Division, Lawrence Berkeley National Laboratory, Berkeley, California, United States of America; 5 United States Department of Energy Joint Genome Institute, Walnut Creek, California, United States of America; Lund University, Sweden

## Abstract

**Background:**

AMP-activated protein kinase (AMPK) is a heterotrimeric enzyme that is evolutionarily conserved from yeast to mammals and functions to maintain cellular and whole body energy homeostasis. Studies in experimental animals demonstrate that activation of AMPK in skeletal muscle protects against insulin resistance, type 2 diabetes and obesity. The regulatory γ_3_ subunit of AMPK is expressed exclusively in skeletal muscle; however, its importance in controlling overall AMPK activity is unknown. While evidence is emerging that gamma subunit mutations interfere specifically with AMP activation, there remains some controversy regarding the impact of gamma subunit mutations [1]–[3]. Here we report the first gain-of-function mutation in the muscle-specific regulatory γ_3_ subunit in humans.

**Methods and Findings:**

We sequenced the exons and splice junctions of the AMPK γ_3_ gene (*PRKAG3*) in 761 obese and 759 lean individuals, identifying 87 sequence variants including a novel R225W mutation in subjects from two unrelated families. The γ_3_ R225W mutation is homologous in location to the γ_2_R302Q mutation in patients with Wolf-Parkinson-White syndrome and to the γ_3_R225Q mutation originally linked to an increase in muscle glycogen content in purebred Hampshire Rendement Napole (RN^-^) pigs. We demonstrate in differentiated muscle satellite cells obtained from the *vastus lateralis* of R225W carriers that the mutation is associated with an approximate doubling of both basal and AMP-activated AMPK activities. Moreover, subjects bearing the R225W mutation exhibit a ∼90% increase of skeletal muscle glycogen content and a ∼30% decrease in intramuscular triglyceride (IMTG).

**Conclusions:**

We have identified for the first time a mutation in the skeletal muscle-specific regulatory γ_3_ subunit of AMPK in humans. The γ_3_R225W mutation has significant functional effects as demonstrated by increases in basal and AMP-activated AMPK activities, increased muscle glycogen and decreased IMTG. Overall, these findings are consistent with an important regulatory role for AMPK γ_3_ in human muscle energy metabolism.

## Introduction

AMP-activated protein kinase (AMPK) is a serine/threonine kinase that is evolutionarily conserved from yeast to mammals and functions as a highly responsive energy sensor in cells [4]–[7]. It acts both centrally and peripherally to coordinate multiple inputs from various hormones, peptides and nutrients to maintain overall energy homeostasis. In response to cellular stress in the periphery, AMPK inhibits anabolic pathways and stimulates catabolic pathways to restore cellular energy charge. *In vitro* and experimental animal studies have demonstrated that altered activity of AMPK has significant implications for a number of cardiovascular risk factors, including type 2 diabetes [4]–[6]. In skeletal muscle, AMPK activation stimulates glucose uptake, fatty acid uptake and oxidation, and mitochondrial biogenesis [5]. Thus AMPK activation in skeletal muscle protects against the development of insulin resistance, type 2 diabetes and obesity [8]–[11].

**Figure 1:**
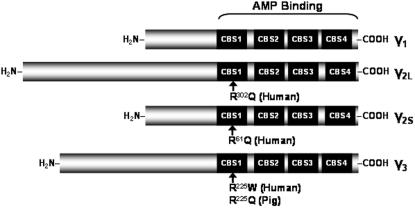
γ subunit isoforms showing the 4 tandem repeats of CBS domains responsible for AMP and ATP binding. Locations of the human R225W and the porcine R225Q mutations in the γ_3_ subunit as well as the R302Q and R61Q mutations in the γ_2_ long and short forms, respectively, are indicated. Figure adapted from [12].

AMPK has a heterotrimeric structure and is composed of an α-catalytic, a β-regulatory and a γ-regulatory subunit. The α-catalytic subunit has two isoforms (α_1_, α_2_), the β-regulatory subunit has two isoforms (β_1_, β_2_), and the γ-regulatory subunit has three isoforms (γ_1_, γ_2_, γ_3_) [12], [13]. Figure 1 depicts the three γ isoforms and their structural characteristics. The various isoforms of each subunit are encoded by distinct genes, with alternative promoters and splice variants, adding to genetic and post-translational complexities [5]. Most isoforms are expressed to some extent in all tissues, although mRNA expression data indicate that the γ_3_ subunit is more abundant in skeletal muscle than in any other tissue [14]. The relative proportions of the γ subunits in human skeletal muscles are as yet unknown. Several exercise studies in humans have measured γ subunit protein levels in the *vastus lateralis* muscle. In these studies, protein levels were expressed as percentage of sedentary controls, pretraining values, or untrained muscles as opposed to absolute relative values [15]–[17]. A more recent study provided evidence that only 3 out of the 12 possible AMPK complex combinations are expressed in human *vastus lateralis*: α_1_/β_2_/γ_1_, α_2_/β_2_/γ_1_ and α_2_/β_2_/γ_3_ [18] . The only heterotrimer shown to be phosphorylated and activated during high intensity exercise was the complex containing the γ_3_ isoform. The activity associated with the other two heterotrimers either remained unchanged or decreased during exercise [18]. The γ_2_ long and short isoforms (γ_2L_ and γ_2S_, respectively) result from alternative splicing of a common transcript in heart and other tissues [19]–[21]. While both long and short isoforms are expressed in heart, γ_2S_ is predominant in skeletal muscle [19].

To date the only published mutations in the AMPK genes in humans are in the γ_2_-regulatory subunit, the predominant γ isoform in cardiac muscle. While AMPK activity stimulates fatty acid oxidation, glucose uptake and glycolysis in the heart, its regulation is complex, and still not well understood [2], [3], [22]. At least six different missense mutations within the CBS domains of γ_2_ have been shown to cause the excess storage of glycogen in pronounced glycogen-associated vacuoles[23]. This excess storage of glycogen results in a complex phenotype, including Wolf-Parkinson-White (WPW) syndrome, conduction system disease and cardiac hypertrophy [21], [24], [25]. In purebred Hampshire Rendement Napole (RN) pigs, a naturally occurring R225Q mutation in the γ_3_ subunit of AMPK was identified and linked to increased glycogen storage in skeletal muscle [26] and to increased resynthesis of glycogen after exercise when R225Q AMPKγ_3_ is overexpressed in transgenic mice [7].

In the context of our ongoing goal of identifying genes that underlie predisposition for diabetes and obesity, we searched for genetic variation in human *PRKAG3*. Of the 24 novel coding variants identified, we focused on the R225W mutation due to the established importance of the mutation in porcine AMPK γ_3_ and the homologous mutations in the γ_2_ subunit of human AMPK. We determined the functional significance of this mutation in differentiated muscle cells from the *vastus lateralis* of R225W carriers compared to matched controls.

Here we describe a previously unreported R225W mutation in the human AMPK γ_3_ gene, *PRKAG3*, found in two independent kindreds. We show that the R225W mutation causes increased basal and AMP-activated AMPK activity, as well as increased muscle glycogen storage and decreased intramuscular triglyceride (IMTG).

## Methods

### Subjects and Sequence Analysis

The coding exons and splice junctions of the human AMPK γ_3_ gene (NCBI accession no. NM_017431) were resequenced in obese Caucasian subjects from the Ottawa Hospital Weight Management Clinic and in lean healthy Caucasian individuals who participated in a study of leanness at the University of Ottawa Heart Institute. The cohorts consisted of 761 obese subjects with a mean BMI of 39.9 kg/m^2^ and>90^th^ percentile adjusted for age and sex, and 759 lean subjects with a mean BMI of 20.1 kg/m^2^ and<25^th^ percentile adjusted for age and sex. The study was approved by the institutional review boards of the University of Ottawa Heart Institute and the Ottawa Hospital. Informed written consent was obtained from all participants. A total of 58 genes were sequenced as part of this study and detailed findings have been presented elsewhere [27]. Selection criteria for our patient population as well as methods for DNA isolation, sequencing and data analysis have been described previously [28].

**Figure 2:**
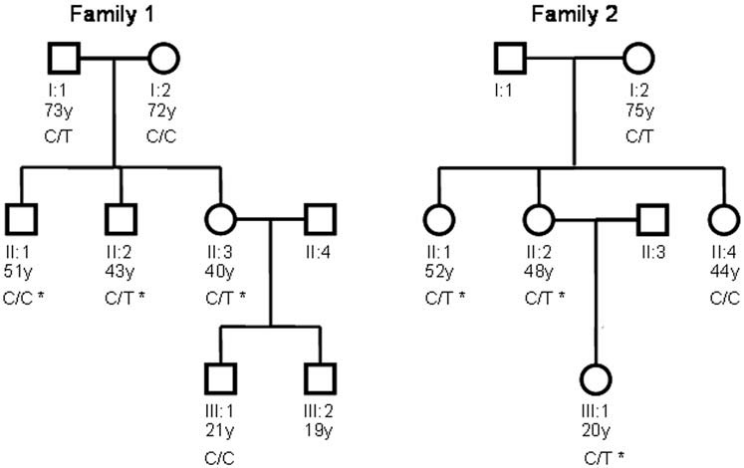
Families of the two probands with the PRKAG3-R225W mutation. *Subjects who underwent muscle biopsy.

Predictions of the possible functional implications of the variants were made using PolyPhen (http://genetics.bwh.harvard.edu/pph/) and SIFT (http://blocks.fhcrc.org/sift/SIFT.html) programs [29], [30]. The R225W variant, which is found in the second CBS domain (Figure 1), was identified to be functionally damaging by both programs. This variant was intriguing since it is analogous to the R225Q mutation causing increased muscle glycogen content in pigs, as well as having the same amino acid location as the mutations in the human γ_2_ subunit that are known to alter AMPK protein function and cause Wolf-Parkinson-White syndrome. We thus contacted subjects that were identified as carrying the R225W mutation in the initial study cohorts, and screened their available family members for further study (Figure 2). The region of the AMPKG3 gene with the R225W variation was sequenced in DNA samples from family members to determine whether they carried the same variation. Subject characteristics are described in Table 1.

**Table 1:** Characteristics of PRKAG3-R225W subjects and controls who underwent muscle biopsy.

	R225W (n = 5)	Controls (n = 5)
Age (years)	40.6±12.4	42.8±12.9
Weight (kg)	82.5±23.2	80.9±27.1
BMI (kg/m^2^)	29.1±9.0	29.2±10.3
Total Cholesterol (mmol/L)	5.54±0.70	4.52±0.66
Body Fat (%)	34.6±10.4	35.6±8.7
Basal Metabolic Rate (kJ)	6773.8±1201.1	5380.4±2134.9
HDL-C (mmol/L)	1.29±0.26	1.52±0.70
LDL-C (mmol/L)	3.22±0.47	2.55±0.35
TC:HDL	4.40±0.71	3.44±1.24
Triglycerides (mmol/L)	2.27±1.00	1.11±0.61
Glucose (mmol/L)	4.94±0.61	5.16±0.63
Insulin (pmol/L)	54.2±10.1	47.4±35.8
HbA1C	0.0522±0.0031	0.0544±0.0013

Blood chemistry measurements were conducted following an overnight fast. Means +/− SD. n = 5, Student's *t-*test, no significant differences between R225W and control subjects.

### Skeletal Muscle Biopsies and Cell Culture

Five subjects with the R225W mutation and five control subjects were matched for gender, age, weight, BMI, as well as amount and intensity of self-reported weekly physical activity. These subjects refrained from physical activity for three days prior to undergoing additional studies in the post-absorptive state after an overnight fast. Percutaneous skeletal muscle biopsies of the *vastus lateralis* muscle were obtained from R225W carriers and their matched controls using a 5 mm Bergstrom needle (Opitek) under local anaesthesia with 1% lidocaine. *Vastus lateralis* is commonly selected for studies of human muscle metabolism and was chosen for biopsy in the current study due to the demonstrated importance of the γ_3_ subunit in this muscle [18]. A 10 mg sample of the biopsy was flash frozen in O.C.T. compound for muscle fiber typing, glycogen content determination and IMTG determination. A 25 mg sample of tissue was subjected to trypsin digestion and cells were grown to ∼70% confluency in Ham's F10 media (supplemented with 1% antibiotic-antimycotic, 2.5 µg/mL gentamycin, 12.4% FBS, 0.41 mg/mL BSA, 826 nM dexamethazone, 8.3 ng/mL epidermal growth factor, 0.41 mg/mL fetuin, and 25 pmol insulin). Isolation of satellite cells was achieved via immuno-sorting using primary anti-5.1H11 (Developmental Studies Hybridoma Bank) and secondary MACS goat anti-mouse IgG microbeads. After the suspension was passed through a magnetic field, labelled cells were isolated, eluted and then grown up in culture. Upon reaching 80% confluency, cells were differentiated for ∼10 days in Dulbecco's modified Eagle's medium (supplemented with 2% horse serum, 1% antibiotic-antimycotic and 2.5 µg/mL gentamycin).

### AMPK Isolation

Approximately 10 days after differentiation, myotubes were incubated on ice while shaking in the presence of lysis buffer (30 mM HEPES, 2.5 mM EGTA, 3 mM EDTA, 70 mM KCl, 0.1% Nonidet P-40, 20 mM β-glycerophosphate, 20 mM NaF, 2 mM NaPPi, 1 mM Na_3_VO_4_, 200 µM PMSF and 1 µM pepstatin A). Partial purification of AMPK from the cell lysate was carried out as detailed in [31], [32]. The cell lysate was incubated at a 1∶1 ratio with 30% polyethylene glycol 6000 for 1 hour. Resulting purified protein was dissolved in assay buffer (62.5 mM Na HEPES, 62.5 mM NaCl, 62.5 mM NaF, 6.25 mM Na pyrophosphate, 1.25 mM EDTA, 1.25 mM EGTA, 1 mM dithiothreitol, 1 mM PMSF, 5 µg/mL SBTI and 1 µM pepstatin A) and then quantified using the bicinchoninic assay.

### AMPK Activity Determination

The SAMS peptide assay was carried out as detailed in [32], [33] with minor modifications: 13 µL reaction buffer (20 mM HEPES-NaOH, 0.4 mM dithiothreitol, 0.01% Brij-35 with or without 300 µM AMP), 10 µL of 562 µM SAMS substrate peptide, 10 µCi [^32^P]ATP (3000 Ci/mmol), and 6 µg of partially purified AMPK were incubated with constant shaking at 30°C for 15 minutes. Negative controls consisted of all reagents except SAMS substrate peptide and AMP. All reactions were carried out in triplicate. AMPK activity was expressed as pmol of ^32^P-radiolabeled phosphate incorporated into the SAMS peptide per minute per mg of protein.

### Quantitative RT-PCR Analysis

RNA from muscle biopsy samples was extracted using Trizol (Invitrogen) and treated with DNaseI (Ambion). cDNA was generated from 0.5 µg of RNA using random decamer primers (Ambion) with MMLV reverse transcriptase (Invitrogen). Amplicons of AMPKG3 and GAPDH genes were cloned into pCR 2.1-TOPO (Invitrogen). Serial dilutions of these constructs were used to generate standard curves, following assessment of primer efficiency and analysis of melting curve profiles. Quantitative real-time PCR analysis was performed in a LightCycler system (Roche) using FastStart DNA MasterPLUS SYBR Green I kit (Roche). The primers for real-time PCR were designed using LightCycler Probe Design Software v1.0 (Roche). Following 30 cycles of amplification with 1.0 µl of cDNA and 0.5 µM of F and R primers per reaction, expression of AMPKG3 was normalized to expression of GAPDH. Primer sequences: F: 5′-CAGAGGACACTATGTCTGG-3′ and R: 5′-GCTTGGGATTGAGGACT-3′ (AMPKG3), F: 5′-GATTTGGCCGTATTGGG-3′ and R: 5′-TCCACGACATACTCAGC-3′ (GAPDH).

### Western Blotting and Densitometry

To determine protein levels of the γ_3_ isoform, the same purified protein extracts that were used for the AMPK activity assay were subjected to immunoblotting. Electrophoresis was carried out using a 12% polyacrylamide gel. Following transfer to nitrocellulose membranes, primary antibodies were incubated at a 1∶1000 dilution overnight at 4°C (AMPKγ_1_, AMPKγ_3_ and β-actin antibodies were from Cell Signaling Technology, Danvers, MA). The secondary antibody was a peroxidase-conjugated goat anti-rabbit IgG (Santa Cruz Inc., Santa Cruz, CA) and was incubated at a 1∶500 dilution for one hour at room temperature. For detection, blots were processed using enhanced chemiluminescence kits (Amersham Pharmacia; Baie d'Urfe, QC, Canada). β-actin was used as the loading control and densitometry was carried out to determine relative protein levels (values expressed as γ isoform integrated densitometry value (IDV) divided by β-actin IDV).

### Muscle Glycogen Content Determination

Glycogen content was determined using Periodic Acid Schiff (PAS) staining. Frozen tissues were cut as 10 µm cross sections and mounted on Superfrost slides. After Schiff staining, sections were counterstained with Harris's hematoxylin. Imaging software (Northern Eclipse) was used to analyze tissues at 200× magnification with an Axiophot digital light microscope (Axiophot 2, Zeiss, Oberkochen, Germany). 2–3 fields of view in each of 3 sections per patient were analyzed for glycogen content as a proportion of surface area of each field.

### Intramuscular Triglyceride (IMTG) Content

lMTG content was determined using osmium tetroxide (OsO_4_) staining. Frozen tissues were cut as 10 µm cross sections and mounted on Superfrost slides and fixed in 10% neutral buffered formalin. IMTG was stained black with 1% OsO_4_ and then cells were counterstained with hematoxylin and eosin (H&E). Imaging software (Northern Eclipse) was used to analyze tissues at 200× magnification with an Axiophot digital light microscope (Axiophot 2, Zeiss, Oberkochen, Germany). 2–3 fields of view in each of 3 sections per patient were analyzed for IMTG content as a proportion of surface area of each field.

### Fiber Typing

Frozen tissues were cut as 10 µm cross sections and mounted on Superfrost slides. Sections were stained using the Vectastain ABC-AP kit (Vector Laboratories). Type 1 fibers were identified by using A4.840 as the primary antibody (Developmental Studies Hybridoma Bank), which is specific to the C-terminal part of the beta slow myosin heavy chain in type I skeletal muscle fibers. Biotinylated goat anti-mouse IgG (BA-9200, Vector Laboratories) was used as the secondary antibody, and then cells were stained red using SK-5100 (Vector Laboratories). Type 2a fibers were identified by using N2.261 as the primary antibody (Developmental Studies Hybridoma Bank), which is specific to the N-terminal part of the beta slow myosin heavy chain in adult skeletal myosin heavy chain type IIA. BA-9200 was used as the secondary antibody, and then cells were stained blue using SK-5300 (Vector Laboratories). Type 2b/x fibers were identified as unstained cells. Fiber type ratios were determined by counting numbers of each fiber type in 2–3 fields of view in 3 sections per patient.

### Statistical Analysis

**Figure 3:**
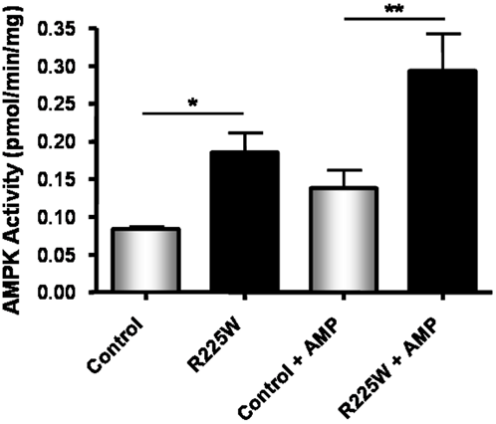
Basal and AMP-activated AMPK activities in differentiated skeletal muscle cells from R225W carriers and matched control subjects. Means +/− SD. n = 3, One-way ANOVA, Tukey post-test, *p = 0.015, **p = 0.001.

**Figure 4:**
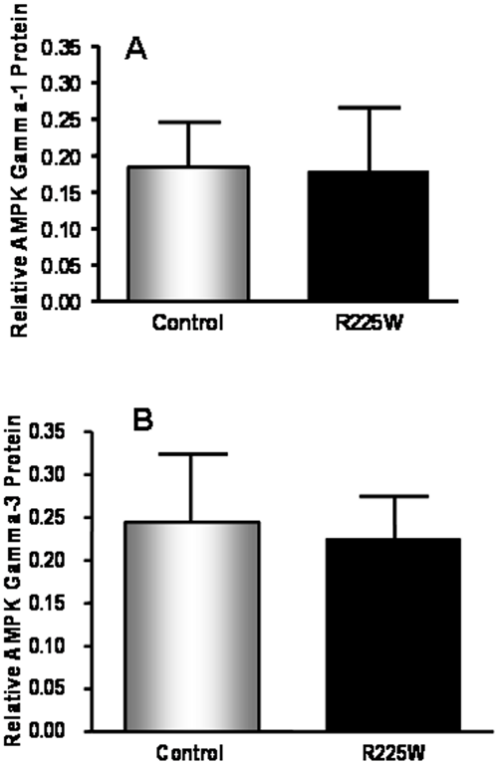
Relative protein levels of the γ_1_ (A) and γ_3_ (B) isoforms in differentiated skeletal muscle cells from R225W carriers and matched control subjects. The integrated densitometry value (IDV) for each γ subunit was divided by the IDV of the loading control, β-actin. Means +/− SD. n = 2–4, Student's *t*-test, p = 0.389 (A), p = 0.229 (B).

The management of clinical and genotypic data was carried out with SAS statistical software (Version 9.1, SAS Institute Inc., Cary, NC). A one-way ANOVA with Tukey post-test was used to determine differences in AMPK activity (Figure 3). Student's *t* tests were used to assess mRNA levels, protein levels, glycogen content, intramuscular triglyceride and fiber type ratio (Figures 4–7).

**Figure 5 pone-0000903-g005:**
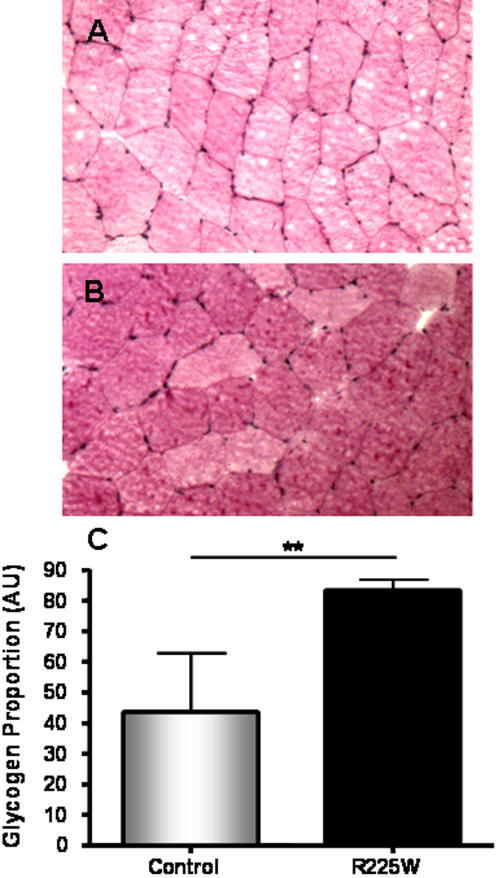
Glycogen content of *vastus lateralis* for R225W and control subjects. Muscle sections were prepared and stained with PAS, as described in the methods section (A: control, B: R225W). C: quantification of glycogen content via imaging software. Means +/− SD. n = 5, Student's *t*-test, **p = 0.002.

**Figure 6 pone-0000903-g006:**
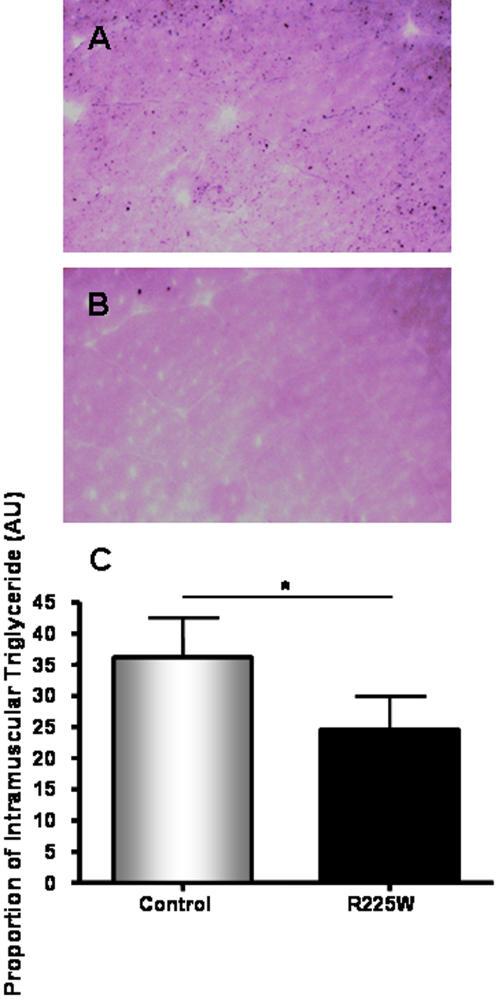
Intramuscular triglyceride content of *vastus lateralis* for R225W and control subjects. Muscle sections were prepared and stained with OsO_4_, as described in the methods section (A: control, B: R225W). C: quantification of IMTG via imaging software. Means +/− SD. n = 4, Student's *t*-test, *p = 0.032.

**Figure 7:**
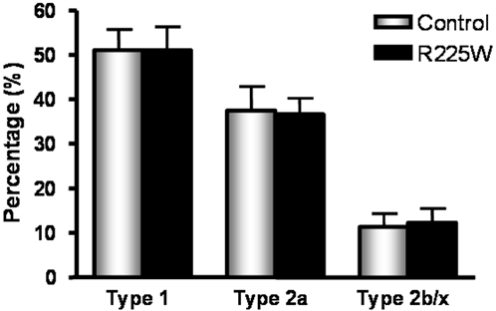
Fiber type ratios of *vastus lateralis* for R225W versus control subjects, expressed as percentage of each fiber type . Means +/− SD. n = 5, Student's *t-*test for each fiber type, no significant differences between R225W and control subjects (Type 1: p = 0.992, Type 2a: p = 0.896, Type 2b/x: p = 0.834).

## Results

### AMPK γ_3_ Sequence Variants

We sequenced the exons and intron/exon boundaries of *PRKAG3*, which codes for the subunit of AMPK that is specific to skeletal muscle. We studied 761 obese subjects with a mean BMI of 39.9 kg/m^2^ and>90^th^ percentile adjusted for age and gender, and 759 lean subjects with a mean BMI of 20.1 kg/m^2^ and<25^th^ percentile adjusted for age and gender. We identified 87 genetic variants in *PRKAG3* and its flanking regions. Of the six common (minor allele frequency>0.05) variants, three were intronic polymorphisms (IVS2+45, IVS6+31 and IVS10+85), one was a synonymous variant (G180G), and two were non-synonymous variants (P71A and R340W). The 81 rare variants included one SNP in the upstream region, one in the 5′ UTR, 50 intronic SNPs, 10 synonymous and 16 nonsynonymous coding variants, one frameshift mutation that leads to a stop codon and two SNPs in the 3′ UTR. The frequency and functional prediction for the coding variants are outlined in Table 2. Of the 30 coding *PRKAG3* variants observed in this study, 24 were not listed in the current SNP database (dbSNPbuild126). Computational predictions of the severity of missense substitutions were made using PolyPhen [29] and SIFT [30].

**Table 2:** Coding variants in the PRKAG3 gene observed in this study.

Variant	Allele	MAF (Obese)	MAF (Lean)	S/NS	Functional Prediction	
					PolyPhen	SIFT
E70E	G>A	0	0.001	S	-	-
P71A (rs692243)	C>G	0.179	0.160	NS	possibly damaging	tolerated
E76Q	G>C	0	0.001	NS	benign	tolerated
E85E	G>A	0.001	0	S	-	-
T87T	C>T	0.003	0.003	S	-	-
D103G	A>G	0.001	0	NS	benign	tolerated
V107fs	indel	0.001	0	frameshift	stop codon	stop codon
G113V	G>T	0	0.001	NS	possibly damaging	tolerated
F139F (rs12328317)	C>T	0.001	0.001	S	-	-
L153V (rs35050588)	C>G	0.001	0	NS	benign	tolerated
L161P	T>C	0.001	0	NS	benign	tolerated
G171S	G>A	0.001	0	NS	benign	tolerated
P179P	C>T	0.001	0	S	-	-
G180G (rs35658907)	C>T	0.026	0.034	S	-	-
G180S	G>A	0	0.001	NS	benign	tolerated
Y194Y	C>T	0.001	0	S	-	-
M197T	T>C	0.001	0	NS	possibly damaging	affected
E211Q	G>C	0.001	0	NS	benign	tolerated
R225W	C>T	0.001	0.001	NS	probably damaging	affected
R225Q	G>A	0	0.001	NS	benign	tolerated
Q260R	A>G	0.001	0.006	NS	benign	tolerated
I269T	T>C	0.001	0	NS	possibly damaging	affected
R307C	C>T	0.001	0	NS	probably damaging	affected
P313P	G>A	0.001	0.001	S	-	-
R340W (rs33985460)	C>T	0.053	0.056	NS	probably damaging	affected
R340Q	G>A	0.001	0	NS	benign	tolerated
R446M	G>T	0	0.001	NS	possibly damaging	tolerated
A482V (rs34720726)	C>T	0.002	0	NS	benign	affected
D485N	G>A	0.005	0.001	NS	benign	tolerated
L487L	C>T	0.001	0	S	-	-

(MAF: minor allele frequency; S/NS: synonymous/nonsynonymous)

Given the established importance of homologous mutations in Rendement Napole (RN^-^)pigs (γ3R225Q) [26], [34], [35] and in the γ_2_ subunit (R302Q/R61Q) in human patients [21], we pursued the functional significance of the novel R225W mutation identified in two unrelated subjects. A total of 11 subjects from the two families were screened for the R225W mutation. Two family members in the first family and three family members in the second family were observed to carry the mutation (Figure 2). Other sequence variants that are predicted to have functional implications according to PolyPhen and SIFT are shown in Table 2. Of interest are variants M197T, I269T, R307C and R340Q, all of which occur at similarly low frequencies in our cohorts.

### Characteristics of R225W and Matched Control Subjects

The five carriers of the R225W variant were matched with control subjects on the basis of gender, age, weight, and BMI. The R225W carriers and control subjects were also well-matched for amount and intensity of self-reported weekly physical activity. Two brothers of the proband in the first family, and the sister and daughter of the proband in the second family consented to muscle biopsies and were included in the study (Figure 2). No significant differences were noted in fasting values for plasma lipids, glucose, insulin,or HbA1C in this small cohort (Table 1). During a hyperinsulinemic glucose clamp study, the rate of glucose infusion required to maintain euglycemia in the last hour of the clamp was somewhat higher in the 2 carriers of the R225W variant as compared to 2 closely matched controls but did not reach a nominal standard of significance (7.3±0.8 *vs*. 5.4±2.4 ml/kg/min, p = 0.16).

### Basal and AMP-Activated AMPK Activity

We determined that the R225W mutation had an overall effect on AMPK activity. Myocytes derived from *vastus lateralis* biopsies were immunopurified and differentiated into myotubes, following which total AMPK activity was assayed using the ‘gold-standard’ SAMS peptide assay [32], [33] in purified protein extracts. Results demonstrated that the basal (p = 0.015) as well as the AMP-activated (p = 0.001) AMPK activities in subjects with the R225W mutation were two-fold higher compared to that found in controls (Figure 3). Control subjects exhibited a 0.054 pmol/min/mg mean increase in activity upon AMP-stimulation, whereas R225W carriers showed a mean increase of 0.108 pmol/min/mg, representing a 65% and a 58% increase from basal activity, respectively. Although R225W carriers demonstrate twice the AMP-stimulated AMPK activity of control subjects, the similar percent increases indicate that R225W AMPK is likely activated by AMP with a similar magnitude to control AMPK. R225W AMPK is therefore not constitutively active or AMP-independent, as it is able to be further activated by AMP.

### Expression of AMPK γ Isoforms

AMPKγ_3_ mRNA levels in skeletal muscle biopsy samples were detected by RT-PCR analysis. There was no difference in AMPKγ_3_ mRNA levels between R225W carriers and control subjects (p = 0.083, 2-tailed t-test). AMPKγ_1_ and γ_3_ protein levels were detected by Western blotting in the same purified protein extracts as used for the AMPK activity assay. β-actin was used as the loading control and densitometry was carried out to determine relative protein levels. There were no differences in either AMPKγ_1_ or γ_3_ protein levels between R225W carriers and control subjects (p = 0.389 and p = 0.229, respectively) (Figure 4a and b). Importantly, the only three complexes that normally contribute to AMPK activity in human *vastus lateralis* are α_1_β_2_γ_1_, α_2_β_2_γ_1_ and α_2_β_2_γ_3_ [18]. We believe that the R225W variant is affecting AMPK activity without affecting the level of transcription or translation of the *PRKAG3* gene.

### Skeletal Muscle Glycogen Content

To determine the effect of the R225W mutation on skeletal muscle glycogen content, we used Periodic Acid Schiff (PAS) staining of cryo-sectioned *vastus lateralis* muscle. As shown in Figure 5, muscle of R225W carriers had approximately 90% more muscle glycogen content than controls (p = 0.002). This result was expected as the Hampshire RN^-^ pigs carrying the R225Q γ_3_AMPK mutation have increased muscle glycogen content [26].

### Intramuscular Triglyceride (IMTG) Content

We hypothesized that IMTG would be reduced in skeletal muscle of subjects with the R225W variant, given observations in transgenic mice expressing the homologous mutation in the AMPK γ_3_ gene and fed a high fat diet [7]. Osmium tetroxide (OsO_4_) staining of sections of the *vastus lateralis* muscle indicated lower IMTG levels in the R225W carriers as compared to matched controls (p = 0.032) (Figure 6).

### *Vastus Lateralis* Muscle Fiber Type Composition

Finally, we determined whether or not there were differences in muscle fiber type composition in the biopsied muscle. Immunohistochemistry of the sections revealed a similar proportional distribution of fiber types in subjects with the R225W mutation compared to controls (Figure 7). Fiber type ratio is an important measure due to its influence on muscle glycogen content and IMTG content. Since there are no differences in fiber type ratio between R225W subjects and controls, differences in glycogen and IMTG cannot be attributed to fiber type composition.

## Discussion

AMPK is widely recognized as a key metabolic regulator of energy expenditure in peripheral tissues and of energy intake in the hypothalamus. In this study we focused on the functional importance of a rare mutation in *PRKAG3* encoding the AMPK regulatory γ_3_ subunit that is expressed exclusively in skeletal muscle. While the functions of the three AMPK subunits have not been fully characterized, it is becoming increasingly clear that the γ subunit is a major regulator of holoenzyme activity [4]–[6], [22]. The γ subunit is thought to be involved in AMP binding, and in the regulation of glycogen content as variants in its CBS domains affect glycogen metabolism in skeletal and cardiac muscle [19], [23], [25], [26], [36]. This is the first report of functional effects of nonsynonymous sequence variants in the γ_3_ subunit in humans. We have demonstrated that the R225W mutation increases basal and AMP-activated AMPK activities in differentiated muscle cells obtained from the *vastus lateralis* of carriers (Figure 3), and that γ_1_ and γ_3_ protein levels are unchanged in R225W carriers (Figure 4). Normally in human *vastus lateralis*, the complexes that contribute to AMPK activity are α_1_β_2_γ_1_, α_2_β_2_γ_1_ and α_2_β_2_γ_3_ [18]. Future studies will need to assess the subunit combinations that contribute to AMPK activity in R225W carriers. We have also demonstrated that R225W results in increased skeletal muscle glycogen content (Figure 5) and decreased IMTG content (Figure 6), while there was no change in muscle fiber type composition (Figure 7).

Further studies are required to characterize the effects of R225W on metabolic responses to challenges such as acute and chronic exercise. However, given the literature on Hampshire RN^-^ pigs with an analogous γ_3_ mutation [26], [34], [35], and the Tg-R225Qγ_3_ mice [37], [38], it is anticipated that the human R225W variant would result in improved capacity during chronic exercise challenges due to their increased muscle glycogen. The natural occurrence of mutations in AMPKγ_3_ was first discovered in Hampshire RN^-^ pigs [26], [34]. The single missense mutation, arginine 225 to glutamine (R225Q) results in a dominant mutation that is homologous to the γ_3_ R225W variant described here. R225Q causes an approximate 70% increase in skeletal muscle glycogen, but no change in heart and liver glycogen content, resulting in poor meat quality. Other phenotypic effects include lower muscle fat content and increased oxidative capacity. Furthermore, Hampshire RN^-^ pigs carrying the mutation showed normal resting levels of glucose and insulin as well as normal glucose tolerance tests. When challenged with exercise, it was found that R225Q pigs depleted their glycogen stores at normal rates, but re-synthesized glycogen at faster rates than control pigs due to increased muscle glucose uptake rates [34], [35]. This limited evidence indicated no functional impairments associated with the increased glycogen in skeletal muscle. Our findings that human R225W carriers have an approximate 90% increase in skeletal muscle glycogen, an approximate 30% decrease in intramuscular triglyceride, and normal levels of plasma lipids, glucose, insulin and HbA1C are consistent with the animal studies described above. Future studies will investigate the physiological response of these subjects to exercise challenges.

Recently mice bearing the homologous human AMPK γ_3_ transgene, Tg-R225Q, were developed to study the effects of the naturally occurring pig mutation. The R225Q mutation produced a fully dominant protein [7] with increased AMPK activity. ACC phosphorylation was increased in the basal state as well as after stimulation with AICAR (an artificial activator of AMPK), indicating that the mutated AMPK could be activated [7]. Glycogen content of various muscles in mice bearing the R225Q mutation have 1.5–2 times the glycogen content of controls [36], consistent with our findings in humans. Moreover, exercise increased ACC phosphorylation and decreased intramuscular triglyceride (IMTG) in the *gastrocnemius* muscle of transgenic, compared to wild-type, mice. Tg-R225Q mice thus oxidized more fat in the AMP-stimulated state (during exercise) compared to wild-type controls [38]. As demonstrated in the current study, the R225Wγ_3_ mutation in humans is a gain-of-function mutation that exhibits increased basal and AMP-activated activity of AMPK. It should be noted that total cellular AMPK activity was assayed in this study, not immuno-purified γ_3_ activity. Therefore, the R225W mutation in γ_3_ has an important effect overall on total AMPK activity in skeletal muscle. It will be of interest in future studies to determine whether the α_1_β_2_γ_1_, α_2_β_2_γ_1_ and α_2_β_2_γ_3_ subunit combinations normally found in human *vastus lateralis* are also formed in skeletal muscle of R225W carriers.

Interestingly, this novel mutation is analogous to the γ_2_ subunit mutation that causes inherited cardiac arrhythmias and cardiac hypertrophy [21], [24], [25]. While it is known that AMPK activity stimulates fatty acid oxidation, glucose uptake and glycolysis in the heart, its regulation is complex, and still not well understood [22]. Missense mutations within the CBS domains of γ_2_ have been shown to cause the excess storage of glycogen in pronounced glycogen-associated vacuoles [23]. This excess storage of glycogen is thought to result in myocyte hypertrophy, and gives rise to WPW syndrome and cardiac conduction disease [23]. The enhanced skeletal muscle glycogen deposition demonstrated in carriers of the analogous γ_3_ mutation is consistent with the above results in the heart. Subsequent introduction of some of the γ_2_ mutations into the AMPK homologue in yeast (SNF1/SNF4) produced a constitutively active enzyme, indicating that these heterozygous missense mutations were dominant in their effect [25]. Our findings indicate that the γ_3_ R225W mutation increases basal AMPK activity, but results in a protein that can be further activated by AMP, unlike the latter γ_2_ mutations.

In summary, we report the identification of human subjects bearing a R225W mutation in *PRKAG3* which causes increased basal and AMP-stimulated AMPK activity, increased glycogen content and decreased IMTG in skeletal muscle. These findings are relevant to an improved understanding of the regulatory role that AMPK plays in skeletal muscle energy metabolism. The γ_3_ subunit represents an attractive pharmacological target due to its exclusive expression in skeletal muscle. It has already been demonstrated that Metformin, commonly prescribed for treatment of type 2 diabetes, acts via stimulation of AMPK [39]–[41]; and although it is not possible to draw conclusions regarding therapeutics from this study, it is tempting to hypothesize that the stimulation of AMPK activity in skeletal muscle alone, without affecting AMPK activity in the brain or heart, could be effective in the treatment of obesity and type 2 diabetes mellitus.
